# Diagnostic significance of cardiac bridging integrator 1 score in hospitalized patients with heart failure with preserved ejection fraction and its assessment of prognostic value for major adverse cardiac events

**DOI:** 10.1186/s12872-025-05399-9

**Published:** 2025-12-10

**Authors:** Xia Guo, Haoxuan Chu, Hanchi Xu, Zhen Guo, Yulin Tian, Shipeng Wang, Yushi Wang

**Affiliations:** Department of Cardiovascular Medicine, The First Hospital of Jilin University, Changchun, 130021 China

**Keywords:** Bridging integrator 1, CS score, Heart failure with preserved ejection fraction, Major adverse cardiac events, Biomarker

## Abstract

**Background:**

Cardiac bridging integrator 1 (cBIN1) is a cardiomyocyte-specific protein critical for excitation–contraction coupling. The cBIN1 score (CS), derived from plasma cBIN1 levels, serves as a non-invasive biomarker reflecting myocardial microstructural integrity and is unaffected by systemic inflammation or BMI.

**Methods:**

A total of 108 HFpEF patients and 108 matched controls were retrospectively included. All subjects underwent clinical evaluation, echocardiography, and biochemical testing. Plasma cBIN1 was measured by ELISA, and CS was calculated. Multivariate logistic regression and ROC analyses were used to assess the diagnostic and prognostic value of CS for HFpEF and major adverse cardiac events (MACE) during 1-year follow-up. A sensitivity analysis was performed by excluding patients with chronic kidney disease or eGFR < 60 ml/min/1.73 m² to address renal-related confounding.

**Results:**

CS levels were significantly higher in the HFpEF group (*p* < 0.001). LVEF, E/e′, neutrophil-to-lymphocyte ratio (NLR), BNP, and CS were independently associated with HFpEF. CS showed positive correlations with BNP, sST2, left atrial diameter, and E/e′, and a negative correlation with LVEF. ROC analysis yielded an AUC of 0.805 for CS in diagnosing HFpEF (sensitivity 70.4%, specificity69.4%, cutoff = 3.902). Diagnostic performance improved when CS was combined with BNP (AUC = 0.953) or sST2 (AUC = 0.834). During follow-up, patients with CS ≥ 3.902 had significantly increased MACE risk (OR = 4.318, *p* = 0.002), establishing CS as an independent prognostic marker. The sensitivity analysis confirmed these findings remained robust: CS retained independent diagnostic value for HFpEF and predictive value for MACE, with stable diagnostic performance of CS alone and in combination with BNP/sST2, and minimal variation in optimal cutoff.

**Conclusion:**

CS is an independent diagnostic marker for HFpEF and predictor of MACE, correlating with established cardiac markers and echocardiographic parameters. Its diagnostic accuracy is significantly enhanced when combined with BNP or sST2, supporting its clinical utility in early diagnosis and risk stratification in HFpEF.

**Supplementary Information:**

The online version contains supplementary material available at 10.1186/s12872-025-05399-9.

## Introduction

 Heart failure with preserved ejection fraction (HFpEF) has become one of the predominant forms of heart failure globally [1]. Driven by an aging population and the rising prevalence of metabolic disorders, the incidence of HFpEF continues to increase and is expected to impose a growing clinical and economic burden over the coming decade [2]. Currently, more than 50% of heart failure cases are attributed to HFpEF [3], which is primarily characterized by pronounced diastolic dysfunction and an elevated risk of arrhythmias. Unlike heart failure with reduced ejection fraction (HFrEF), HFpEF is characterized by a heterogeneous pathophysiology involving structural cardiac remodeling, microvascular dysfunction, systemic inflammation, oxidative stress, comorbid conditions, and neurohormonal activation [4]. Patients with HFpEF commonly present with ventricular hypertrophy and structural cardiac remodeling, resulting in impaired diastolic function and elevated filling pressures. Microvascular dysfunction further compromises myocardial perfusion, exacerbating symptoms. In parallel, systemic inflammation and oxidative stress contribute to cardiomyocyte injury, while comorbidities such as hypertension, diabetes, and obesity not only increase HFpEF risk but also accelerate disease progression [3, 5]. Concurrent activation of the sympathetic nervous system and the renin–angiotensin–aldosterone system increases cardiac workload and promotes fluid retention [6]. Collectively, these mechanisms impair cardiac function, adversely affecting quality of life and prognosis in HFpEF patients [7, 8]. Notably, the interplay between microvascular dysfunction and inflammation is considered central to HFpEF pathogenesis, potentially driving abnormal myocardial energy metabolism and fibrotic remodeling [5]. HFpEF is associated with diverse clinical manifestations, including reduced exercise tolerance, dyspnea, and peripheral edema. Notably, older adults with HFpEF often present with atypical symptoms such as fatigue, cognitive changes, and delirium, which are less common in younger patients [9].

Cardiac-specific circulating biomarkers, such as brain natriuretic peptide (BNP) family and cardiac troponin, remain essential for the diagnosis and prognostic evaluation of HFpEF [10]. However, elevated natriuretic peptide levels are not exclusive to heart failure and may also result from other cardiac conditions (e.g., acute coronary syndrome, myocarditis) and non-cardiac factors (e.g., advanced age, anemia, obesity, renal dysfunction). Notably, a study by Wang et al. involving 3,389 participants demonstrated a negative correlation between obesity and BNP/NT-proBNP levels [11]. As natriuretic peptide concentrations are closely tied to fluid status, distinguishing HFpEF from non-cardiac volume overload, such as in renal failure, can be challenging. This limitation is particularly pronounced in elderly individuals and those with impaired renal function. Therefore, additional biomarkers capable of reflecting intrinsic myocardial health—independent of volume status, inflammation, comorbidities, or lifestyle factors—are needed to enhance diagnostic accuracy in HFpEF. This need is primarily limited to HFpEF due to its key differences from other heart failure subtypes (e.g., HFrEF): First, HFpEF is pathologically driven by diastolic dysfunction, myocardial microstructural remodeling, and high heterogeneity (frequent comorbidities like obesity) [12, 13], unlike HFrEF (with established systolic dysfunction). Second, existing biomarkers (e.g., BNP) are often confounded by comorbidities in HFpEF [14] and fail to reflect intrinsic myocardial health, while HFrEF has a mature biomarker system. Third, biomarkers like the cBIN1 score (CS)—which targets myocardial microstructures—fill a unique gap in HFpEF, a need less urgent in other subtypes [15, 16].

Cardiac bridging integrator 1 (cBIN1) plays a crucial role in maintaining normal excitation–contraction coupling and calcium handling in cardiomyocytes, with its expression closely linked to myocardial structural remodeling [17]. Clinical studies have demonstrated that reduced cBIN1 levels are significantly associated with heart failure (HF), highlighting its potential as a novel diagnostic and therapeutic target [18]. Beyond its role in calcium cycling, cBIN1 also contributes to the electrical stability of cardiomyocytes, thereby potentially mitigating malignant arrhythmias during HF progression. Recent research indicates that cBIN1 microdomains in T-tubules are shed into the peripheral circulation via microvesicles, facilitating a steady-state turnover. This supports the use of circulating cBIN1 as a liquid biopsy biomarker for evaluating myocardial health. Based on this concept, the CS—a dimensionless inverse index derived from plasma cBIN1 concentrations—has been developed. CS demonstrates promising utility for the diagnosis and prognostic assessment of HF patients [19]. A key distinction between this study and Nikolova et al. (2018) is that our data were obtained from hospitalized HFpEF patients, who are likely to experience more severe fluid overload, leading to higher BNP levels. This difference is crucial, as it impacts the interpretation of BNP as a diagnostic and prognostic marker in HFpEF. In contrast, Nikolova et al. studied outpatients, where BNP levels might not reflect the same acute fluid overload conditions seen in hospitalized patients. Recent preclinical studies have investigated gene therapy approaches to restore or enhance cBIN1 expression in cardiomyocytes, aiming to improve T-tubule integrity and calcium handling [16]. In isoproterenol-induced heart failure models, adeno-associated virus (AAV)-mediated cBIN1 gene transfer effectively preserved T-tubule architecture, enhanced calcium transients, and improved myocardial contractility, thereby delaying—or even reversing—the progression of heart failure [20, 21]. These findings offer promising insights into the development of precision therapies targeting HFpEF.

## Materials and methods

### Study design

This study enrolled patients diagnosed with HFpEF at the Department of Cardiology, First Hospital of Jilin University, between June 2023 and January 2024. The control group comprised healthy individuals without cardiovascular risk factors. HFpEF diagnosis was established based on objective evidence of fluid overload in the absence of non-cardiac causes, confirmed by senior heart failure specialists. Eligible patients had a documented history of heart failure hospitalization or invasive hemodynamic evidence of elevated filling pressures. Individuals with an LVEF < 50% at enrollment were excluded. A total of 108 patients were included in each group. Follow-up was conducted at 1, 3, 6, and 12 months post-discharge to monitor the incidence of major adverse cardiac events (MACE). The study was approved by the institutional ethics committee. The CS was calculated as the natural logarithm of the inverse plasma cBIN1 concentration. Details of plasma processing and storage are described in the Methods section. Baseline clinical, laboratory, and echocardiographic data were extracted from the hospital’s electronic medical records. The Human BIN1 ELISA kit was purchased from Wuhan Fine Biotech Co., Ltd. (Product Code: EH2422, 96 T kit).

### Sample processing and CS determination

Fasting venous blood samples were collected from all enrolled patients within 24 h of admission. Part of the samples was sent to the Laboratory Department of the First Hospital of Jilin University for biochemical testing, including B-type natriuretic peptide (BNP), serum creatinine, and soluble suppression of tumorigenicity 2 (sST2). The remaining samples were obtained from the Cardiology Department’s biobank and transferred to the hospital’s Public Experimental Platform for further analysis. Plasma was isolated by centrifugation at 2,500 rpm for 15 min at 2–8 °C. The supernatant was aliquoted into 2 ml EP tubes, uniformly labeled, and stored at − 80 °C to preserve sample integrity. All procedures were conducted under strict quality control to avoid repeated freeze–thaw cycles and ensure the reliability of test results.

The concentration of cBIN1 in plasma was measured using a double-antibody sandwich enzyme-linked immunosorbent assay (ELISA). A standard curve was generated in Excel using cBIN1 standard concentrations (x-axis) and corresponding optical density (OD) values (y-axis), with concentration values derived through curve-fitting software. The CS was calculated as: CS = ln(10/cBIN1), where cBIN1 is the measured plasma concentration (pg/mL) [19]. Notably, this formula yields a higher CS value when the plasma cBIN1 concentration is lower, thereby ensuring that the CS score increases with disease severity, which aligns intuitively with conventional biomarker interpretation.

### Definition of MACE events

In this study, MACE were defined as a composite clinical endpoint reflecting significant progression of cardiovascular disease during follow-up at 1, 3, 6, and 12 months post-discharge. MACE included: (1) cardiovascular death—death directly attributable to cardiovascular causes; (2) non-fatal myocardial infarction—clinically and biochemically confirmed acute myocardial infarction not resulting in death; (3) non-fatal stroke—neurological dysfunction due to ischemic or hemorrhagic stroke without fatal outcome; and (4) cardiovascular rehospitalization—hospital readmission due to worsening cardiovascular conditions or complications requiring intervention.

MACE event assessment referred to the literature by Mansoor Husain et al.; [22] causes of death were adjudicated by an independent clinical endpoint committee consisting of three cardiologists, with all available clinical records, laboratory tests, imaging data, and death certificates reviewed [23], and cardiovascular death defined per ACC/AHA criteria [24].

### Follow-up protocol

To ensure data completeness and accuracy, all enrolled patients underwent follow-up assessments at 1, 3, 6, and 12 months post-discharge to capture both early and long-term cardiovascular events. Follow-up was conducted via telephone interviews and outpatient visits. The primary endpoint recorded during follow-up was the occurrence of MACE.

### Statistical analysis

All statistical analyses were performed using R software (version 3.4.3). Continuous variables were expressed as mean ± standard deviation for normally distributed data or as median with interquartile range for skewed data. Categorical variables were presented as counts and percentages. Between-group comparisons of continuous variables were conducted using independent samples t-tests or Mann–Whitney U tests, as appropriate. Categorical variables were compared using the chi-square test or Fisher’s exact test (when expected frequencies < 5). For ordinal data, the Mann–Whitney U test was applied. Multivariate analysis was performed using binary logistic regression. Variable selection was conducted using a forward stepwise method based on maximum likelihood estimation (entry criterion: α = 0.05; removal criterion: α = 0.10). Correlations between continuous variables were assessed using Pearson’s test for normally distributed data or Spearman’s test for non-normal data. Receiver operating characteristic (ROC) curve analysis was used to evaluate the diagnostic performance of the variables. The area under the curve (AUC), sensitivity, specificity, and Youden index were calculated to determine diagnostic accuracy and optimal cutoff values. Odds ratios (ORs) with 95% confidence intervals (CIs) were reported, and a two-tailed *p*-value < 0.05 was considered statistically significant.

### Sensitivity analysis

To address the potential confounding effect of renal dysfunction on BNP levels, we performed a sensitivity analysis by excluding patients who had either a self-reported history of chronic kidney disease (CKD) or an estimated glomerular filtration rate (eGFR) < 60 ml/min/1.73 m² at admission. For this subset of patients, we repeated all primary analyses, including univariate and multivariate logistic regression to identify independent predictors of HFpEF; ROC curve analysis to re-evaluate the diagnostic performance of BNP, CS, and their combination, with determination of the optimal CS cutoff value via the Youden index; and logistic regression to assess the prognostic value of CS and BNP for MACE during follow-up.

## Results

### Study cohorts

A total of 108 patients were included in both the HFpEF and control groups. At baseline, significant differences (*p* < 0.05) were observed between the two groups in diastolic blood pressure, history of myocardial infarction, hypertension, diabetes, atrial fibrillation, chronic kidney disease, stroke, as well as in CS, LVEF, LVDD, IVS, LVPW, left atrial size, E/e′, septal e′ < 7 cm/s, peak tricuspid regurgitation velocity > 2.8 m/s, BNP, sST2, serum sodium, NLR, hematocrit, and uric acid levels. No significant differences were found in the remaining variables. Detailed baseline characteristics are shown in Tables 1 and 2.

**Table 1:** Baseline clinical characteristics and medical history of patients in the HFpEF and control groups

	Total(*n* = 216)	HFpEF group(*n* = 108)	Control groups(*n* = 108)	P值
Age(years)	68.00(61.00,73.00)	69.00(61.25,73.00)	67.00(60.00,72.75)	0.368
Sex				0.892
Male (%)	113(52.3)	56(51.9)	57(52.8)	
Female (%)	103(47.7)	52(48.1)	51 (47.2)	
BMI (kg/m2)	25.23(22.60,27.99)	24.73(21.80,27.85)	25.63(23.40,28.04)	0.068
SBP (mmHg)	133.00(120.00,145.00)	136.50(120.00,152.00)	130.00(120.50,141.00)	0.050
DBP (mmHg)	80.00(72.00,87.00)	76.00(68.25,85.00)	83.00(75.00,89.00)	< 0.001
Heart rate (bpm)	76.00(70.00,89.00)	75.50(68.00,85.00)	78.00(71.00,90.75)	0.135
History of prior myocardial infarction				0.009
Yes (%)	57(26.4)	37(34.3)	20(18.5)	
No %)	159(73.6)	71(65.7)	88(81.5)	
Hypertension				0.020
Yes (%)	157(72.7)	86(79.6)	71(65.7)	
No (%)	59(27.3)	22(20.4)	37(34.3)	
Atrial fibrillation				< 0.001
Yes (%)	32(14.8)	30(27.8)	2(1.9)	
No (%)	184(85.2)	78(72.2)	106(98.1)	
Chronic kidney disease				0.003
Yes (%)	18(8.3)	15(13.9)	3(2.8)	
No (%)	198(91.7)	93(86.1)	105(97.2)	
Stroke				< 0.001
Yes (%)	39(18.1)	32(29.6)	7(6.5)	
No (%)	177(81.9)	76(70.4)	101(93.5)	
Diabetes				0.040
Yes (%)	95(44.0)	55(50.9)	40(37.0)	
No (%)	121(56.0)	53(49.1)	68(63.0)	

Continuous variables are described as weighted mean ± standard deviation (SD), while categorical variables are presented as unweighted n (%)

*Abbreviations*: *BMI* body mass index, *SBP* Systolic Blood Pressure, *DBP* Diastolic Blood Pressure, *HFpEF* Heart Failure with Preserved Ejection Fraction

**Table 2:** Comparison of CS, admission echocardiographic parameters, and laboratory findings between the HFpEF and control groups

	Total(*n* = 216)	HFpEF group(*n* = 108)	Control groups(*n* = 108)	P值
CS	3.90 (3.76,4.14)	4.13(3.89,4.33)	3.80(3.67,3.94)	< 0.001
LVEF (%)	63.00(58.00,64.00)	58.00(55.25,63.00)	63.00(63.00,65.00)	< 0.001
LVDD (mm)	47.00(45.00,50.00)	49.00(44.00,53.00)	47.00(45.00,49.00)	0.002
IVS (mm)	9.00(8.00,10.00)	10.00(8.00,11.00)	8.50(8.00,9.00)	< 0.001
LVPW (mm)	9.00(8.00,10.00)	9.00(8.00,10.00)	8.00(8.00,9.00)	< 0.001
Left atrial diameter (mm)	37.36 ± 6.76	41.23 ± 6.74	33.49 ± 4.02	< 0.001
E/e’				< 0.001
>14(%)	89(41.2)	81(75.0)	8(7.4)	
<14(%)	127(58.8)	27(25.0)	100(92.6)	
E/A				0.116
>1(%)	162(75.0)	86(79.6)	76(70.4)	
<1(%)	54(25.0)	22(20.4)	32(29.6)	
Septal e′ velocity < 7 cm/s				< 0.001
Yes (%)	15(70.8)	90(83.3)	63(58.3)	
No (%)	63(29.2)	18(16.7)	45(41.7)	
Peak tricuspid regurgitation velocity > 2.8 m/s				< 0.001
Yes (%)	35(16.2)	33(30.6)	2(1.9)	
No (%)	181(83.8)	75(69.4)	106(98.1)	
BNP (pg/ml)	87.00(53.00,187.75)	187.50(112.75,338.00)	56.00(43.25,78.00)	< 0.001
eGFR (ml/min/1.73m2)	90.00(71.03,110.00)	87.50(71.03,109.00)	93.27(71.21,110.68)	0.403
sST2 (ng/ml)	11.00(8.00,20.00)	17.00(11.00,31.00)	9.00(7.00,11.00)	< 0.001
LDL-C (mmol/L)	2.35(1.90,3.00)	2.36(1.88,3.00)	2.32(1.91,3.00)	0.727
Serum sodium (mmol/L)	140.00(139.00,142.00)	140.00(137.00,141.00)	141.00(140.00,142.00)	< 0.001
TC (mmol/L)	3.80(3.12,4.75)	3.74(3.03,4.71)	3.91(3.12,4.85)	0.593
NLR	2.29(1.78,3.67)	3.19(2.14,6.10)	2.25(1.53,2.59)	< 0.001
Hematocrit (L/L)	0.42(0.38,0.45)	0.39(0.35,0.42)	0.43(0.41,0.46)	< 0.001
UA (µmol/L)	364.00(305.50,433.75)	387.00(312.25,480.75)	335.00(296.25,403.75)	0.002
Yes (%)	15(70.8)	90(83.3)	63(58.3)	
No (%)	63(29.2)	18(16.7)	45(41.7)	

Continuous variables are described as weighted mean ± standard deviation (SD), while categorical variables are presented as unweighted n (%)

*Abbreviations*:* LVEF* left ventricular ejection fraction, *LVDD *left ventricular end diastolic diameter, *E/e* the ratio of early mitral inflow velocity to early diastolic mitral annular velocity, *NLR* neutrophil to Lymphocyte ratio, *UA* uric acid, *IVS* interventricular septum, *LVPW* left ventricular posterior wall thickness, *TC* total cholesterol, *LDL-C* low density lipoprotein cholesterol, *BNP* B-type natriuretic peptide, *sST2* soluble suppression of tumorigenicity 2, *eGFR* estimated glomerular filtration rate, *CS* Cardiac bridging integrator 1 score, *HFpEF* Heart Failure with Preserved Ejection Fraction

Stepwise logistic regression identified LVEF, LVDD, E/e′, NLR, BNP, and CS as independent predictors of HFpEF. In the final model, LVEF (*p* < 0.001; OR = 0.517, 95% CI: 0.374–0.715), E/e′ (*p* = 0.001; OR = 26.541, 95% CI: 3.731–188.789), NLR (*p* = 0.034; OR = 1.538, 95% CI: 1.033–2.289), BNP (*p* < 0.001; OR = 1.063, 95% CI: 1.027–1.100), and CS (*p* = 0.001; OR = 19.204, 95% CI: 3.492–105.618) were all significantly associated with HFpEF (Table 3).

**Table 3:** Multivariate logistic regression analysis of independent predictors for HFpEF

Variables	B	SE	Wald		*P*	OR	95% CI	
							Lower	Upper
LVEF (%)	-0.660	0.166	15.873		<0.001	0.517	0.374	0.715
LVDD (mm)	0.107	0.081	1.716		0.190	1.113	0.948	1.305
E/e'	3.279	1.001	10.728		0.001	26.541	3.731	188.789
NLR	0.430	0.203	4.493		0.034	1.538	1.033	2.289
BNP (pg/ml)	0.061	0.017	12.145		<0.001	1.063	1.027	1.100
CS	2.955	0.870	11.543		0.001	19.204	3.492	105.618

*Abbreviations*: *LVEF* left ventricular ejection fraction, *LVDD* left ventricular end diastolic diameter, *E/e* the ratio of early mitral inflow velocity to early diastolic mitral annular velocity, *NLR* neutrophil to Lymphocyte ratio, *BNP *B-type natriuretic peptide, *CS *cardiac bridging integrator 1 score, *HFpEF* Heart Failure with Preserved Ejection Fraction

### CS correlation analysis

The CS score was significantly correlated with several cardiac biomarkers and echocardiographic parameters. Specifically, CS showed positive correlations with BNP (*r* = 0.355, *p* < 0.001), sST2 (*r* = 0.290, *p* = 0.002), LVDD (*r* = 0.187,*p* = 0.006), left atrial size (*r* = 0.317, *p* < 0.001), E/e′ (*r* = 0.371, *p* < 0.001), and peak tricuspid regurgitation velocity > 2.8 m/s (*r* = 0.235, *p* = 0.038). In contrast, CS was negatively correlated with LVEF (*r* = − 0.243, *p* < 0.001). No significant correlations were found between CS and IVS, LVPW, septal e′ < 7 cm/s, or the E/A ratio. Detailed results are presented in Table 4.

**Table 4:** Correlation analysis

Variables	*P*	*r*
BNP (pg/ml)	< 0.001	0.355
sST2 (ng/ml)	0.002	0.290
LVEF (%)	< 0.001	−0.243
LVDD (mm)	0.006	0.187
IVS (mm)	0.316	0.068
LVPW (mm)	0.128	0.104
Left atrial diameter (mm)	< 0.001	0.317
E/e’	< 0.001	0.371
Septal e′ velocity < 7 cm/s	0.318	0.068
E/A	0.402	0.057
Peak tricuspid regurgitation velocity > 2.8 m/s	< 0.001	0.235

*Abbreviation*s: *LVEF* left ventricular ejection fraction, *LVDD* left ventricular end diastolic diameter, *E/e* the ratio of early mitral inflow velocity to early diastolic mitral annular velocity, *BNP* B-type natriuretic peptide, *sST2* soluble suppression of tumorigenicity 2, *CS* cardiac bridging integrator 1 score, *IVS* interventricular septum, *LVPW* left ventricular posterior wall thickness

**Fig. 1:**
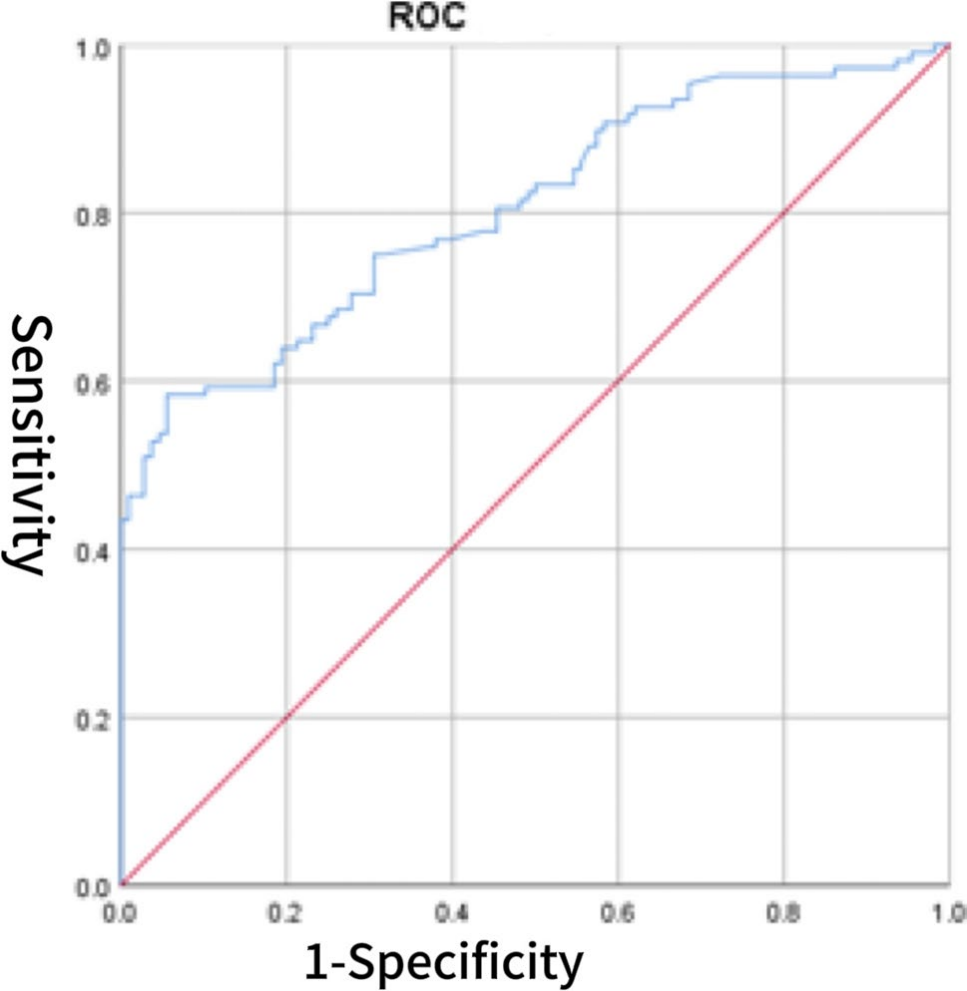
ROC curve of CS for predicting HFpEF

**Fig. 2:**
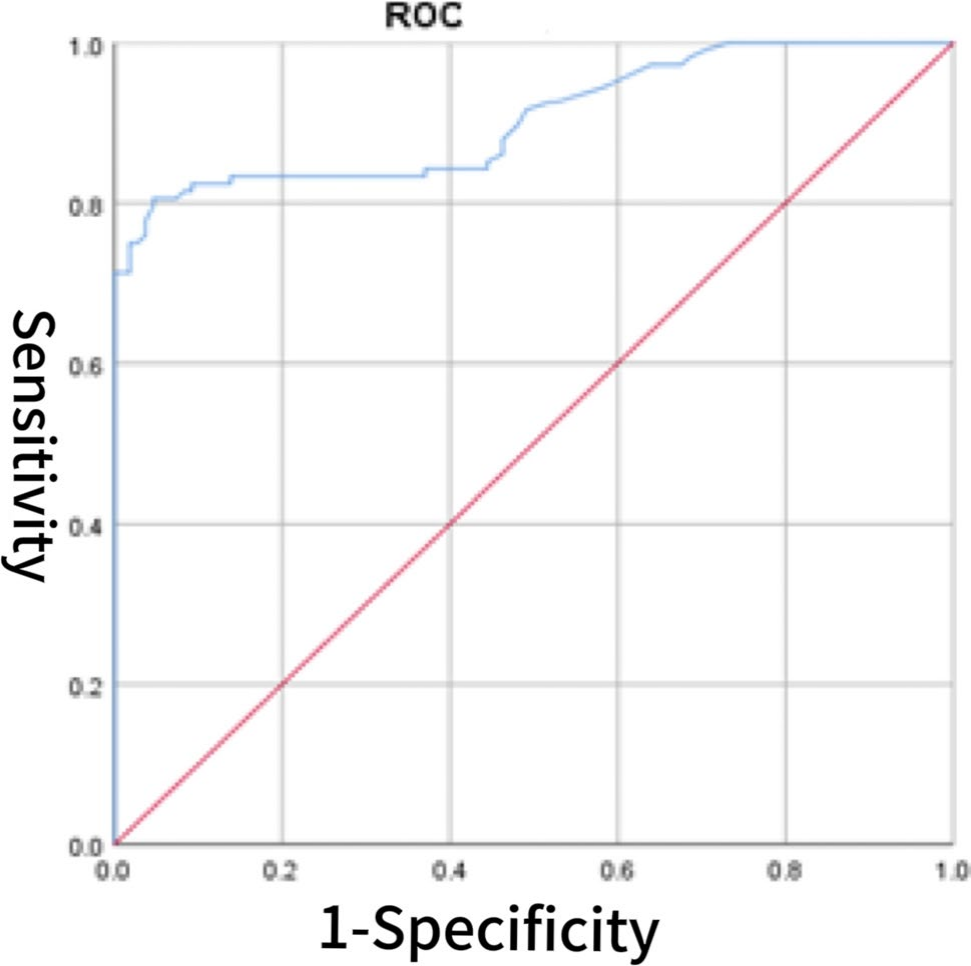
ROC curve of BNP for predicting HFpEF

**Fig. 3:**
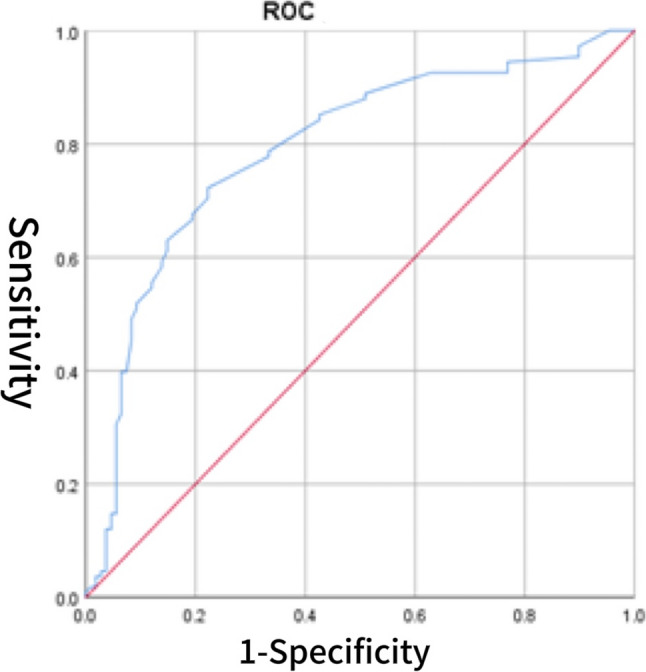
ROC curve of sST2 for predicting HFpEF

**Fig. 4:**
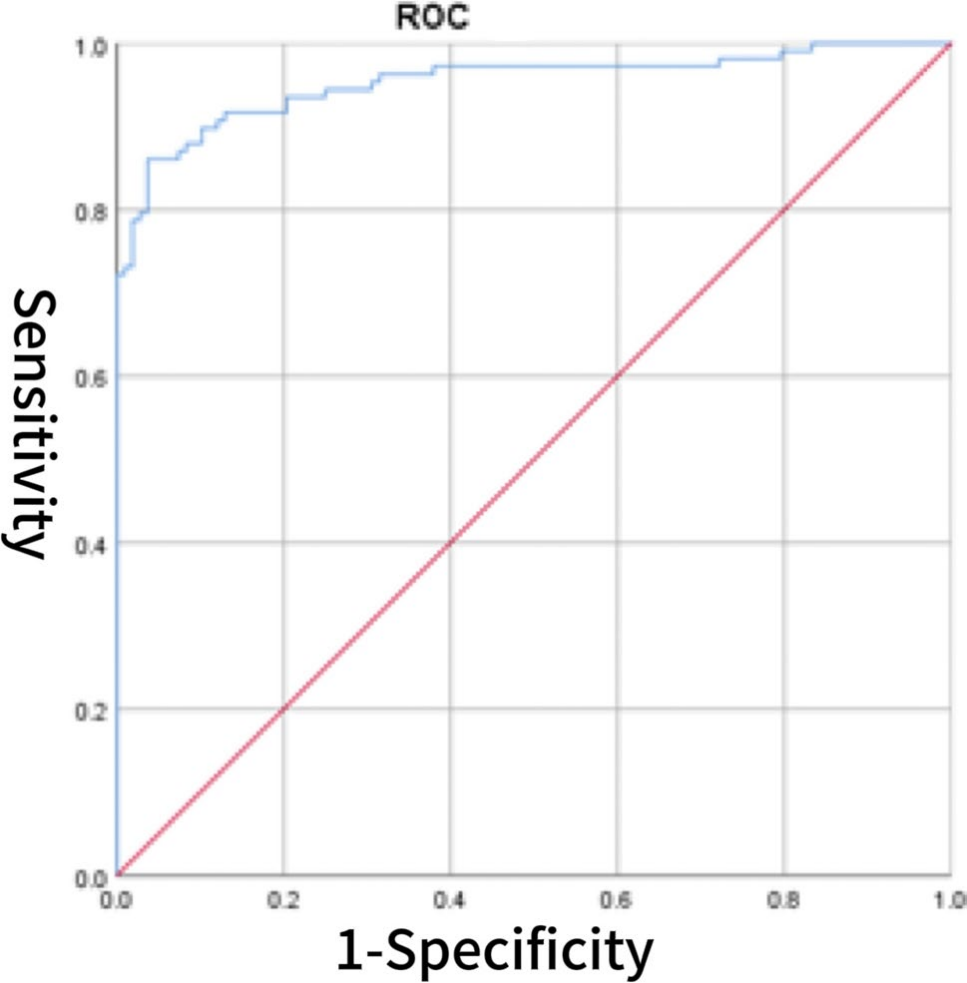
ROC curve of CS combined with BNP for predicting HFpEF

**Fig. 5:**
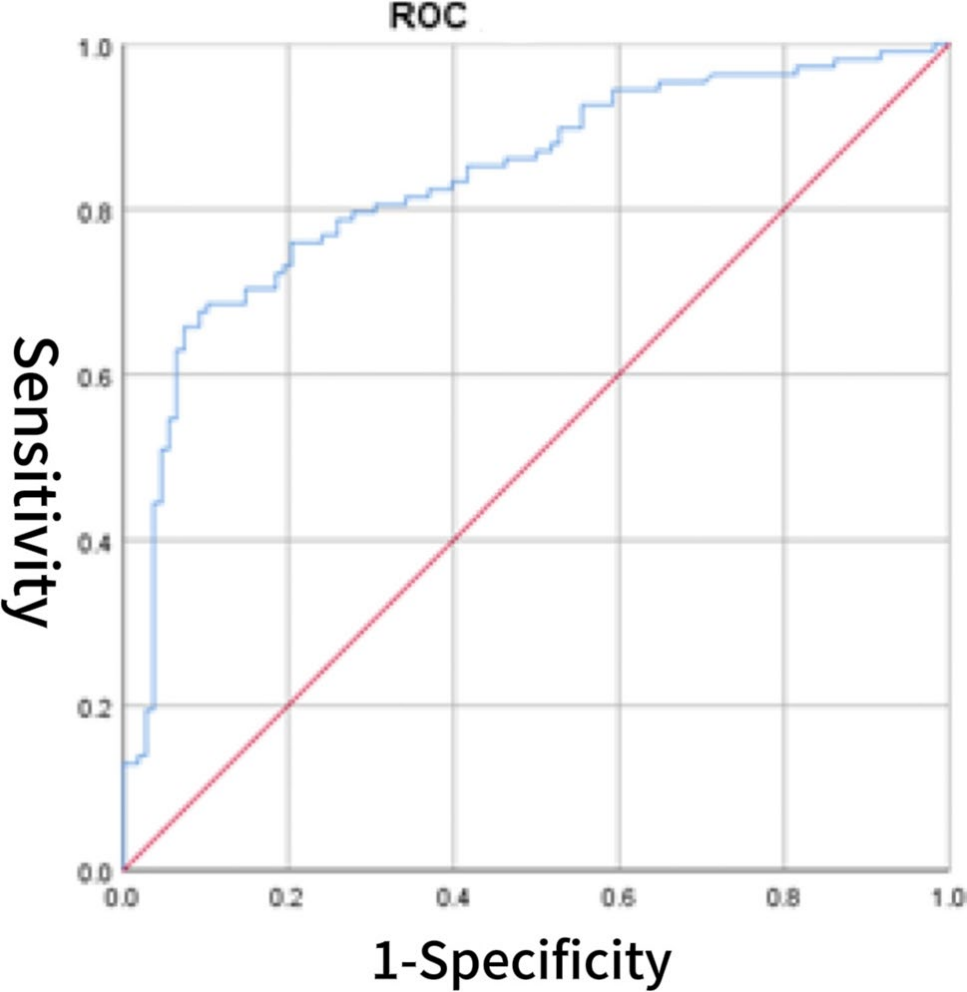
ROC curve of CS combined with sST2 for predicting HFpEF

### The diagnostic value of CS for HFpEF

The diagnostic value of the CS score for HFpEF was evaluated using ROC curve analysis. The area under the AUC for CS alone was 0.805 (95% CI: 0.747–0.863), with an optimal cutoff value of 3.902, yielding a sensitivity of 70.4% and a specificity of 69.4% (Fig. 1). For comparison, BNP demonstrated a higher AUC of 0.905 (95% CI: 0.864–0.946), with a sensitivity of 80.6% and specificity of 95.4% (Fig. 2). The AUC for sST2 was 0.792 (95% CI: 0.730–0.854), with a sensitivity of 72.2% and specificity of 77.8% (Fig. 3). Combining CS with BNP yielded an AUC of 0.953 (95% CI: 0.925–0.982), with a sensitivity of 86.1% and specificity of 96.3% (Fig. 4). The combination of CS and sST2 produced an AUC of 0.834 (95% CI: 0.779–0.889), with a sensitivity of 68.5% and specificity of 89.8% (Fig. 5). These results indicate that while CS alone has moderate diagnostic value for HFpEF, its performance is inferior to BNP. However, combining CS with BNP or sST2 significantly enhances diagnostic accuracy, particularly specificity. See Table 5 for a detailed comparison.

**Table 5:** Diagnostic value of CS, BNP, their combinations, sST2, and CS combined with sST2 for cardiovascular events in HFpEF patients

Variables	AUC	*P*-value	Sensitivity	Specificity	Cutoff value	Youden’s index	95% CI Lower	95% CI Upper
CS	0.805	< 0.001	70.4%	69.4%	3.902	0.398	0.747	0.863
BNP (pg/ml)	0.905	< 0.001	80.6%	95.4%	103.5	0.760	0.864	0.946
CS combined with BNP	0.953	< 0.001	86.1%	96.3%	0.579	0.824	0.925	0.982
sST2(ng/ml)	0.792	< 0.001	72.2%	77.8%	11.300	0.500	0.730	0.854
CS combined with sST2	0.834	< 0.001	68.5%	89.8%	0.533	0.583	0.779	0.889

*Abbreviations*: *BNP* B-type natriuretic peptide, *sST2* soluble suppression of tumorigenicity 2, *CS* cardiac bridging integrator 1 score, *HFpEF* Heart Failure with Preserved Ejection Fraction

### Predictive value of CS cutoff for MACE

To evaluate the predictive utility of the CS score for MACE and identify independent influencing factors, this study conducted follow-up assessments at 1, 3, 6, and 12 months post-discharge. Based on the occurrence of MACE, participants were categorized into a MACE group (*n* = 45) and a non-MACE group (*n* = 165), with 6 patients lost to follow-up. ROC curve analysis identified an optimal CS cutoff value of 3.902, corresponding to a Youden index of 0.398. Accordingly, patients were stratified into two groups: CS < 3.902 and CS ≥ 3.902.

Using MACE occurrence as the dependent variable, variables previously found to be statistically significant were included in the model. After variable selection via stepwise regression, a multivariable logistic regression analysis was performed. The results indicated that patients with CS ≥ 3.902 had a significantly increased risk of MACE compared to those with CS < 3.902 (OR = 6.526, 95% CI: 2.158–19.736, *p* = 0.001). These findings suggest that CS is an independent predictor of MACE and has considerable clinical value for risk stratification in patients with HFpEF. Refer to Table 6 for detailed results.

**Table 6:** Predictive performance of CS for MACE occurrence

Variables	B	SE	Wald	*P*-value	OR	95% CI Lower Upper	
Stratification by CS	1.463	0.476	9.456	0.002	4.318	1.700	10.972
LVEF (%)	−0.126	0.046	7.521	0.006	0.881	0.805	0.965
BNP (pg/ml)	0.008	0.002	21.439	< 0.001	1.008	1.005	1.012

*Abbreviations*: *LVEF* left ventricular ejection fraction, *BNP* B-type natriuretic peptide

### Results of sensitivity analysis

After excluding patients with a history of CKD or an eGFR < 60 ml/min/1.73 m² at admission, 185 patients (96 in the HFpEF group and 89 in the control group) were included in the sensitivity analysis. Multivariate logistic regression further confirmed that LVEF, E/e′, NLR, BNP, and CS remained independent predictors of HFpEF, with odds ratios comparable to those in the full cohort, indicating robust associations in patients with preserved renal function. ROC curve analysis showed that BNP, CS, and sST2 all retained significant diagnostic ability for HFpEF (all *p* < 0.001). The optimal cutoff value for CS in this subset was 3.938 (slightly higher than the original 3.902 in the full cohort), with a sensitivity of 69.7% and specificity of 75%. The combined model maintained superior diagnostic accuracy compared to individual markers, confirming their complementary value. In the HFpEF subgroup with preserved renal function, CS ≥ 3.938 remained significantly associated with an increased risk of MACE during follow-up (OR = 4.011, 95% CI: 1.428–11.261, *p* = 0.008), consistent with the primary findings (Supplementary Tables 2–4 and Supplementary Figs. 1–5).

## Discussion

HFpEF is an important subtype of heart failure defined by preserved LVEF but significant diastolic dysfunction [25]. Its incidence has risen sharply in recent years, particularly among the elderly, due to population aging and the growing prevalence of comorbidities such as hypertension, diabetes, and obesity [26]. However, the diagnosis and treatment of HFpEF remain challenging because of its complex and highly heterogeneous pathophysiology. Current diagnostic markers, such as BNP and LVEF, can be influenced by patient comorbidities and clinical characteristics, limiting their utility, especially in older adults [27]. As a result, there is an urgent need to identify novel biomarkers that directly reflect myocardial microstructural and functional changes, facilitating earlier and more accurate diagnosis, risk stratification, and targeted therapy for HFpEF.

cBIN1 is a membrane scaffold protein primarily localized within the T‑tubules of cardiomyocytes, where it plays a pivotal role in regulating calcium signaling and excitation–contraction coupling. Alterations in its expression and associated mechanistic pathways have emerged as a key area of heart failure research [28–30]. As a member of the BAR-domain protein superfamily, cBIN1 possesses a distinctive N‑terminal BAR domain that interacts with the cell membrane, facilitating membrane curvature and thereby supporting the formation and maintenance of the T‑tubule structure. cBIN1 also interacts with L‑type calcium channels (LTCCs) via its C‑terminal SH3 domain, facilitating their clustering and stabilization within the T‑tubule membrane and thereby enhancing calcium influx and excitation–contraction coupling [31, 32]. These properties underscore its critical role in regulating calcium dynamics and myocardial contractility. In HFpEF, impaired diastolic function is closely associated with abnormal calcium transients in cardiomyocytes [33]. Notably, T‑tubule remodeling and functional disruption have been identified as key contributors to this dysregulation. By anchoring LTCCs and ryanodine receptors within a coupled microdomain, cBIN1 maintains the synchrony of calcium transients, making it an essential mediator of T‑tubule integrity and calcium handling in HFpEF [34]. The heterogeneity of HFpEF may arise from variations in cBIN1 expression across its different etiological subtypes. For example, reduced cBIN1 levels in diabetic cardiomyopathy have been associated with impaired exercise tolerance and worsening glucose metabolism [35]. In hypertensive heart disease, mechanical stress-induced hypertrophy may drive T‑tubule remodeling, further disrupting cBIN1-mediated calcium handling [36]. Moreover, decreased cBIN1 expression can destabilize the electrical properties of cardiomyocytes, increasing the risk of arrhythmias [15, 37]. These findings suggest that cBIN1 expression patterns could form the basis for molecular subtyping of HFpEF, offering potential targets for precision therapy. Consistent with prior studies, our findings demonstrate that cBIN1 levels are significantly lower in HFpEF patients compared with healthy controls, indicating reduced cBIN1 expression in this population. The CS, a blood-based biomarker derived from cBIN1 levels, has been proposed for risk stratification and prognosis in heart failure patients [38]. In the present study, CS was significantly correlated with established cardiac biomarkers such as BNP and sST2, as well as key echocardiographic parameters, including left atrial size and the E/e′ ratio, suggesting its potential clinical utility in the diagnosis and prognostic assessment of heart failure. Moreover, previous studies have identified early T‑tubule structural remodeling as a hallmark of heart failure progression, with such changes closely linked to alterations in cBIN1 expression [39]. Downregulation of BIN1 has been closely linked to the development and progression of heart failure, including both HFpEF and HFrEF [40, 41]. Given the pivotal role of cBIN1 in HFpEF pathophysiology, researchers have explored its therapeutic potential. In animal models, AAV9-mediated delivery of the cBIN1 gene successfully restored its expression in cardiomyocytes, leading to significant improvements in both diastolic and systolic cardiac function [42, 43]. These findings highlight cBIN1 not only as a novel biomarker of cardiomyocyte health in HFpEF, but also as a promising target for early diagnosis and precision therapy.

This study included patients diagnosed with HFpEF at the time of hospitalization. At baseline, there were no significant differences between the HFpEF and control groups in diastolic blood pressure, medical history (prior myocardial infarction, hypertension, diabetes, atrial fibrillation, chronic kidney disease, or stroke), CS, LVEF, LVDD, IVS, LVPW, left atrial diameter, E/e′, septal e′ velocity < 7 cm/s, peak tricuspid regurgitation velocity > 2.8 m/s, BNP, sST2, serum sodium, NLR, hematocrit, or uric acid levels (all *p* > 0.05).

This is the first study to systematically evaluate the diagnostic value of cBIN1 and its association with MACE in patients with HFpEF. Our results demonstrated that CS was significantly higher in the HFpEF group compared with the control group (4.13 vs. 3.80, *p* < 0.001). Moreover, multivariate binary logistic regression confirmed CS as an independent risk factor for HFpEF. Consistent with previous studies in HFrEF, where CS has been established as a biomarker for cardiovascular risk, these findings highlight its potential clinical significance in HFpEF. CS was positively correlated with BNP (*r* = 0.355), sST2 (*r* = 0.290), left atrial size (*r* = 0.317), LVDD (*r* = 0.187), peak tricuspid regurgitation velocity > 2.8 m/s (*r* = 0.235), and E/e′ (*r* = 0.371), and negatively correlated with LVEF (*r* = − 0.243). The underlying mechanism may relate to CS reflecting both the structural integrity of the T‑tubule network and its role in diastolic dysfunction and left ventricular remodeling. These results support CS as a valuable adjunctive biomarker for HFpEF assessment, complementing established markers such as BNP and sST2.

ROC curve analyses confirmed the robust diagnostic performance of the CS for HFpEF, yielding an AUC of 0.805 (95% CI: 0.747–0.863) with a sensitivity of 58.3% and a specificity of 94.4%. These results highlight CS as an effective marker for distinguishing HFpEF patients from healthy controls. Notably, when combined with BNP or sST2, CS significantly improves diagnostic precision. The CS–BNP combination achieved an AUC of 0.953 (95% CI: 0.925–0.982), with a sensitivity of 86.1% and specificity of 96.3%, making it highly effective for differentiating HFpEF from other states associated with fluid overload. In contrast, BNP or sST2 alone demonstrated lower specificity, underscoring the incremental diagnostic value of incorporating CS. The high rates of rehospitalization, mortality, and adverse events associated with HFpEF underscore the clinical importance of accurate risk stratification. In this study, MACE was defined as cardiovascular death, non-fatal myocardial infarction, non-fatal stroke, or cardiovascular rehospitalization. At the one‑year follow‑up, MACE occurred in 21.4% of HFpEF patients, with cardiovascular rehospitalization being the most common event (48.9%), likely reflecting the chronic nature of HFpEF pathology, including myocardial fibrosis, microvascular dysfunction, and hemodynamic disturbances. Importantly, CS emerged as a strong and independent prognostic indicator. ROC analysis identified an optimal CS cutoff of 4.047 (Youden index = 0.527), with CS values ≥ 4.047 associated with a significantly increased risk of cardiovascular rehospitalization (OR = 7.367, 95% CI: 2.341–23.176, *p* = 0.001). These findings support CS as a valuable biomarker for both early HFpEF diagnosis and long‑term risk prediction, offering a powerful tool for guiding clinical management. Sensitivity analysis excluding patients with renal impairment confirmed the robustness of our primary findings. Key associations, including the diagnostic, and prognostic roles of BNP, sST2 and CS, persisted stably, unaffected by renal dysfunction-related confounders. Notably, CS exhibited minimal variability across analyses, underscoring its reliability relative to BNP and sST2 in differentiating cardiac from renal fluid overload.

The findings of this study provide novel insights into the early diagnosis and prognostic evaluation of HFpEF. Plasma cBIN1 measurement enables dynamic monitoring of myocardial structural and functional changes, thereby enhancing diagnostic accuracy and risk stratification. As a non-invasive, easily obtainable biomarker, the CS offers a rapid and practical tool with significant potential for clinical application. When combined with traditional markers such as BNP and sST2, CS further improves diagnostic precision and supports personalized management and prognostic assessment in patients with HFpEF.

Our study advances Nikolova et al. (2018) through three key innovations. Our study builds on the foundational work by Nikolova et al. (2018) through three key innovations. First, whereas prior research focused on stable outpatients, we centered our analysis on hospitalized HFpEF patients—a cohort where short-term risk assessment is clinically urgent. This focus addresses a critical gap in understanding CS utility in acute care settings. Second, we extended beyond the single-biomarker paradigm of earlier studies by investigating CS in combination with BNP and sST2. They provide a more holistic framework for diagnosis and risk prediction than previously explored. Third, we derived CS cutoff values specific to our hospitalized cohort, which differ from those reported in the 2018 outpatient-based study. This work enriches the clinical reference landscape for CS, highlighting the need for context-specific thresholds and offering practical insights for its application in inpatient settings. Together, these contributions strengthen the evidence base for CS in contemporary HFpEF management, even within the constraints of a single-center design.

## Limitations

First, this study is limited by its single-center design and relatively small sample size; therefore, larger multicenter prospective studies are warranted to validate the clinical utility of the CS. Second, the plasma half-life of cBIN1 remains unknown, which may impact its stability and reliability as a biomarker. Furthermore, the precise molecular mechanisms underlying cBIN1’s role in HFpEF pathogenesis have yet to be fully elucidated. Another limitation is that we did not include follow-up blood draws to assess changes in BNP and CS levels during hospitalization or after discharge. Monitoring these biomarkers over time would provide valuable insights into how BNP levels decrease in response to treatment, while CS may remain more stable. Future studies should focus on these aspects to strengthen the theoretical basis for the clinical application of CS, including incorporating follow-up blood samples to examine the dynamics of these biomarkers in HFpEF patients. Finally, a limitation of this study is the lack of data regarding the effects of drugs prescribed for heart failure (HF) on circulating levels of cBIN1. Future studies should consider including information on medication use to evaluate its potential impact on cBIN1 levels and its role in heart failure progression.

## Conclusion

This study demonstrates that the CS, a non-invasive biomarker reflecting myocardial microstructural and functional alterations, provides significant diagnostic and prognostic value in HFpEF. CS independently predicts HFpEF occurrence and MACE. Moreover, combining CS with established biomarkers such as BNP and sST2 enhances diagnostic accuracy and risk stratification. These findings support the clinical utility of CS as a complementary tool for early diagnosis and personalized management of HFpEF.

## Supplementary Information

Supplementary Material 1.

Supplementary Material 2.

Supplementary Material 3.

Supplementary Material 4.

Supplementary Material 5.

Supplementary Material 6.

## Data Availability

Data can be provided by the corresponding author upon reasonable request.
