# Supplementary figures and images for: Diagnostic significance of cardiac bridging integrator 1 score in hospitalized patients with heart failure with preserved ejection fraction and its assessment of prognostic value for major adverse cardiac events
Source: BMC Cardiovasc Disord. 2025 Dec 10;26:35. doi: 10.1186/s12872-025-05399-9 (PMC12801533; doi:10.1186/s12872-025-05399-9)

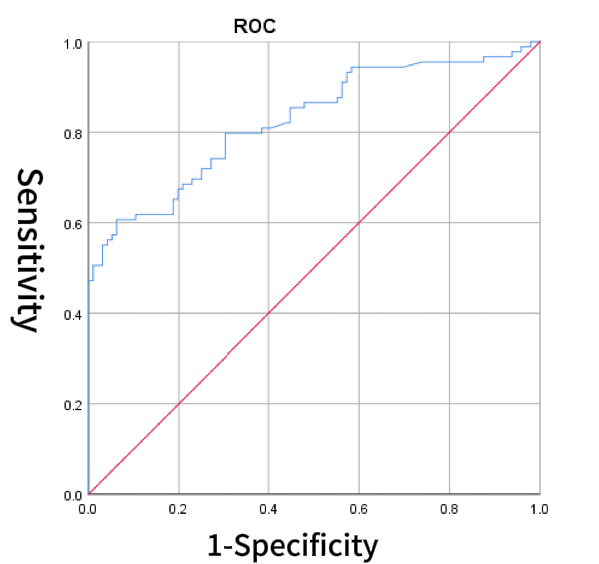

Supplement: Supplementary file 1 — Supplementary Material 1. [file 12872_2025_5399_MOESM1_ESM.tif]

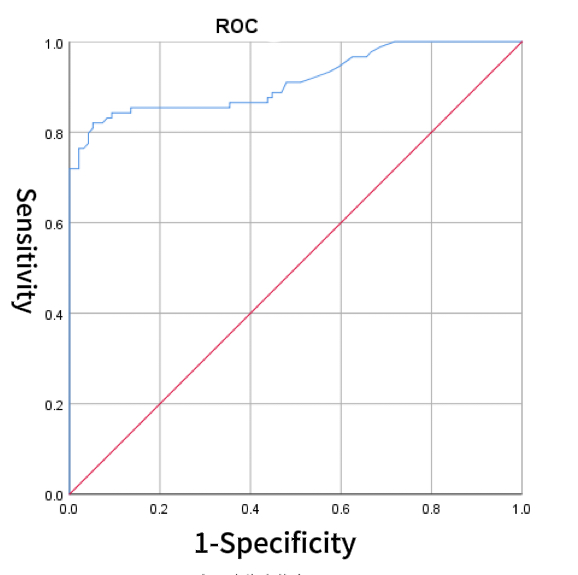

Supplement: Supplementary file 2 — Supplementary Material 2. [file 12872_2025_5399_MOESM2_ESM.tif]

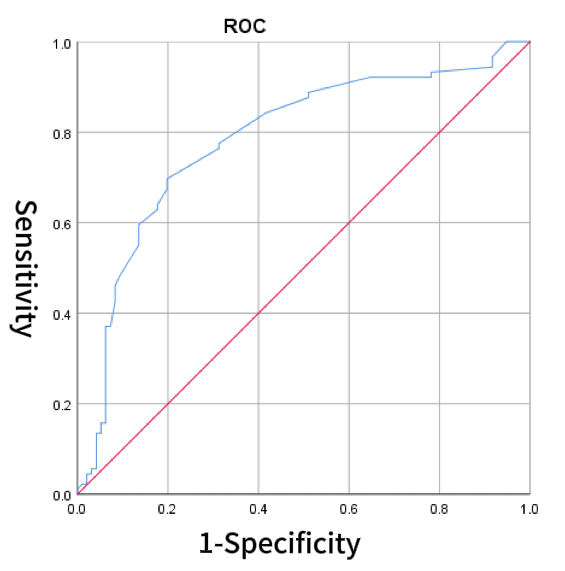

Supplement: Supplementary file 3 — Supplementary Material 3. [file 12872_2025_5399_MOESM3_ESM.tif]

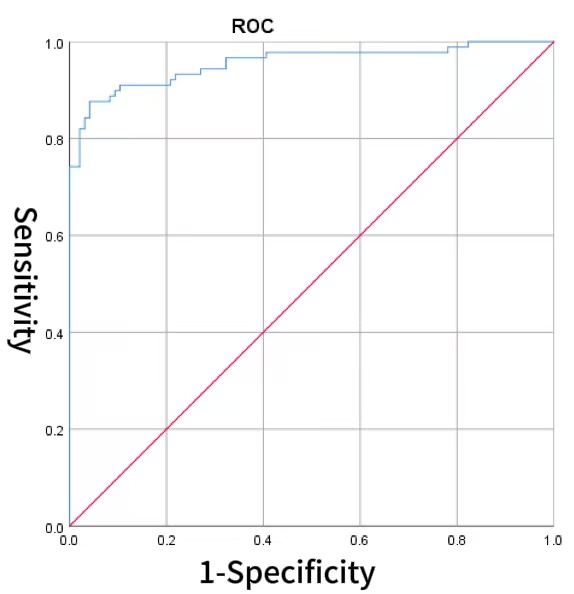

Supplement: Supplementary file 4 — Supplementary Material 4. [file 12872_2025_5399_MOESM4_ESM.tif]

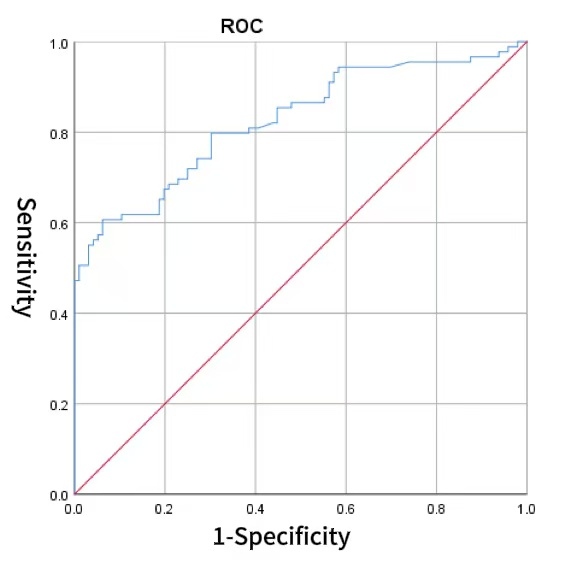

Supplement: Supplementary file 5 — Supplementary Material 5. [file 12872_2025_5399_MOESM5_ESM.tif]
