# Supplementary material for: Diagnostic significance of cardiac bridging integrator 1 score in hospitalized patients with heart failure with preserved ejection fraction and its assessment of prognostic value for major adverse cardiac events
Source: BMC Cardiovasc Disord. 2025 Dec 10;26:35. doi: 10.1186/s12872-025-05399-9 (PMC12801533; doi:10.1186/s12872-025-05399-9)
Supplement: Supplementary file 6 — Supplementary Material 6. [file 12872_2025_5399_MOESM6_ESM.docx]

Table S1. Univariate Logistic Regression Analysis of the Association Between Various Variables and HFpEF.

| **Variables** | **B** | | **SE** | | **Wald** | | ***P*** | | **OR** | | **95% CI**  **Lower Upper** | | |
| --- | --- | --- | --- | --- | --- | --- | --- | --- | --- | --- | --- | --- | --- |
| Age(years) | | 0.002 | | 0.016 | | 0.025 | | 0.875 | | 1.002 | | 0.972 | 1.034 |
| Sex | | -0.037 | | 0.272 | | 0.019 | | 0.892 | | 0.964 | | 0.565 | 1.644 |
| BMI (kg/m2) | | -0.053 | | 0.036 | | 2.124 | | 0.145 | | 0.948 | | 0.883 | 1.018 |
| SBP (mmHg) | | 0.014 | | 0.007 | | 4.147 | | 0.042 | | 1.014 | | 1.001 | 1.028 |
| DBP (mmHg) | | 0.034 | | 0.011 | | 9.003 | | 0.003 | | 1.013 | | 1.008 | 1.017 |
| Heart rate (bpm) | | -0.008 | | 0.009 | | 0.916 | | 0.339 | | 0.992 | | 0.975 | 1.009 |
| History of prior myocardial infarction | | 0.830 | | 0.320 | | 6.720 | | 0.010 | | 1.107 | | 1.038 | 1.179 |
| Hypertension | | 0.712 | | 0.313 | | 5.156 | | 0.023 | | 2.037 | | 1.102 | 3.765 |
| Atrial fibrillation | | -0.345 | | 0.295 | | 1.369 | | 0.242 | | 0.709 | | 0.398 | 1.262 |
| Chronic kidney disease | | 1.731 | | 0.648 | | 7.128 | | 0.008 | | 5.645 | | 1.584 | 20.114 |
| Stroke | | 1.804 | | 1.804 | | 16.510 | | <0.001 | | 6.075 | | 2.544 | 14.505 |
| Diabetes | | 0.568 | | 0.277 | | 4.198 | | 0.040 | | 1.764 | | 1.025 | 3.036 |
| LVEF (%) | | -0.406 | | 0.061 | | 44.155 | | <0.001 | | 0.666 | | 0.591 | 0.751 |
| LVDD (mm) | | 0.097 | | 0.032 | | 9.097 | | 0.003 | | 1.102 | | 1.034 | 1.173 |
| IVS (mm) | | 0.555 | | 0.115 | | 23.369 | | <0.001 | | 1.743 | | 1.391 | 2.183 |
| LVPW (mm) | | 0.671 | | 0.141 | | 22.803 | | <0.001 | | 1.956 | | 1.485 | 2.576 |
| Left atrial diameter (mm) | | 0.267 | | 0.038 | | 50.033 | | <0.001 | | 1.306 | | 1.213 | 1.406 |
| E/e' | | 3.624 | | 0.429 | | 71.242 | | <0.001 | | 37.500 | | 16.163 | 87.003 |
| E/A | | -0.498 | | 0.319 | | 2.447 | | 0.118 | | 0.608 | | 0.325 | 1.134 |
| Septal e′ velocity < 7 cm/s | | -1.273 | | 0.324 | | 15.468 | | <0.001 | | 0.280 | | 0.148 | 0.528 |
| Peak tricuspid regurgitation velocity > 2.8 m/s | | 3.149 | | 0.744 | | 17.933 | | <0.001 | | 1.105 | | 1.035 | 1.179 |
| BNP (pg/ml) | | 0.041 | | 0.006 | | 41.877 | | <0.001 | | 1.042 | | 1.029 | 1.055 |
| eGFR (ml/min/1.73m2） | | -0.005 | | 0.006 | | 0.739 | | 0.390 | | 0.995 | | 0.984 | 1.006 |
| sST2 (ng/ml) | | 0.052 | | 0.013 | | 15.265 | | <0.001 | | 1.054 | | 1.026 | 1.082 |
| LDL-C (mmol/L) | | -0.104 | | 0.142 | | 0.532 | | 0.466 | | 0.902 | | 0.682 | 1.191 |
| Serum sodium (mmol/L) | | -0.192 | | 0.050 | | 14.678 | | <0.001 | | 0.825 | | 0.748 | 0.910 |
| TC (mmol/L) | | -0.103 | | 0.101 | | 1.034 | | 0.309 | | 0.902 | | 0.740 | 1.100 |
| NLR | | 0.526 | | 0.121 | | 18.897 | | <0.001 | | 1.692 | | 1.335 | 2.144 |
| Hematocrit (L/L) | | 0.126 | | 0.257 | | 0.239 | | 0.625 | | 1.134 | | 0.685 | 1.879 |
| UA (μmol/L) | | 0.004 | | 0.001 | | 10.499 | | 0.001 | | 1.004 | | 1.002 | 1.007 |
| CS | | 1.680 | | 0.256 | | 42.955 | | <0.001 | | 5.367 | | 3.247 | 8.871 |

Abbreviations: BMI, body mass index; SBP, Systolic Blood Pressure; DBP, Diastolic Blood Pressure; LVEF, left ventricular ejection fraction; LVDD, left ventricular end diastolic diameter; E/e, the ratio of early mitral inflow velocity to early diastolic mitral annular velocity; NLR, neutrophil to Lymphocyte ratio; UA, uric acid; IVS, interventricular septum; LVPW, left ventricular posterior wall thickness. TC, total cholesterol; LDL-C, low density lipoprotein cholesterol; BNP, B-type natriuretic peptide; sST2, soluble suppression of tumorigenicity 2; eGFR, estimated glomerular filtration rate; CS, Cardiac bridging integrator 1 score; HFpEF, Heart Failure with Preserved Ejection Fraction.

Table S2. Sensitivity Analysis: Univariate Logistic Regression of Associations Between Variables and HFpEF After Excluding Patients with Renal Impairment.

| **Variables** | **B** | | **SE** | | **Wald** | | ***P*** | | **OR** | | **95% CI**  **Lower Upper** | | |
| --- | --- | --- | --- | --- | --- | --- | --- | --- | --- | --- | --- | --- | --- |
| Age(years) | | 0.012 | | 0.017 | | 0.504 | | 0.478 | | 1.012 | | 0.979 | 1.046 |
| Sex | | 0.029 | | 0.295 | | 0.010 | | 0.921 | | 1.030 | | 0.578 | 1.834 |
| BMI (kg/m2) | | -0.039 | | 0.041 | | 0.896 | | 0.344 | | 0.962 | | 0.887 | 1.403 |
| SBP (mmHg) | | 0.008 | | 0.008 | | 1.013 | | 0.314 | | 1.008 | | 0.992 | 1.024 |
| DBP (mmHg) | | 0.046 | | 0.013 | | 9.003 | | 0.003 | | 1.019 | | 1.013 | 1.026 |
| Heart rate (bpm) | | -0.014 | | 0.010 | | 1.790 | | 0.181 | | 0.986 | | 0.967 | 1.006 |
| History of prior myocardial infarction | | 0.659 | | 0.337 | | 3.824 | | 0.051 | | 1.932 | | 0.999 | 3.739 |
| Hypertension | | 0.683 | | 0.331 | | 4.260 | | 0.039 | | 1.980 | | 1.035 | 3.786 |
| Atrial fibrillation | | -0.350 | | 0.321 | | 1.185 | | 0.276 | | 0.705 | | 0.376 | 1.323 |
| Stroke | | 1.961 | | 0.516 | | 14.430 | | <0.001 | | 7.109 | | 2.584 | 19.559 |
| Diabetes | | 0.444 | | 0.298 | | 2.218 | | 0.136 | | 1.559 | | 0.869 | 2.797 |
| LVEF (%) | | -0.368 | | 0.062 | | 35.289 | | <0.001 | | 0.692 | | 0.613 | 0.782 |
| LVDD (mm) | | 0.077 | | 0.033 | | 5.468 | | 0.019 | | 1.080 | | 1.012 | 1.151 |
| IVS (mm) | | 0.516 | | 0.124 | | 17.224 | | <0.001 | | 1.676 | | 1.313 | 2.139 |
| LVPW (mm) | | 0.625 | | 0.150 | | 17.416 | | <0.001 | | 1.868 | | 1.393 | 2.505 |
| Left atrial diameter (mm) | | 0.254 | | 0.040 | | 40.152 | | <0.001 | | 1.290 | | 1.192 | 1.395 |
| E/e' | | 3.539 | | 0.460 | | 59.319 | | <0.001 | | 24.565 | | 15.103 | 74.523 |
| E/A | | -0.485 | | 0.346 | | 1.959 | | 0.162 | | 1.624 | | 0.824 | 3.203 |
| Septal e′ velocity < 7 cm/s | | -1.181 | | 0.345 | | 11.723 | | <0.001 | | 0.115 | | 0.064 | 0.031 |
| Peak tricuspid regurgitation velocity > 2.8 m/s | | 2.854 | | 0.753 | | 14.346 | | <0.001 | | 17.354 | | 3.963 | 75.986 |
| BNP (pg/ml) | | 0.043 | | 0.007 | | 38.316 | | <0.001 | | 1.044 | | 1.030 | 1.059 |
| eGFR (ml/min/1.73m2） | | -0.007 | | 0.007 | | 1.251 | | 0.263 | | 0.993 | | 0.980 | 1.006 |
| sST2 (ng/ml) | | 0.042 | | 0.013 | | 10.994 | | <0.001 | | 1.043 | | 1.017 | 1.069 |
| LDL-C (mmol/L) | | -0.138 | | 0.154 | | 0.803 | | 0.370 | | 0.871 | | 0.643 | 1.179 |
| Serum sodium (mmol/L) | | -0.211 | | 0.058 | | 13.159 | | <0.001 | | 0.810 | | 0.723 | 0.908 |
| TC (mmol/L) | | -0.098 | | 0.110 | | 0.797 | | 0.372 | | 0.907 | | 0.731 | 1.124 |
| NLR | | 0.500 | | 0.121 | | 17.081 | | <0.001 | | 1.649 | | 1.301 | 2.090 |
| Hematocrit (L/L) | | 0.196 | | 0.304 | | 0.416 | | 0.519 | | 1.217 | | 0.670 | 2.210 |
| UA (μmol/L) | | 0.005 | | 0.002 | | 9.614 | | 0.002 | | 1.005 | | 1.002 | 1.008 |
| CS | | 1.854 | | 0.296 | | 39.149 | | <0.001 | | 6.385 | | 3.572 | 11.411 |

Abbreviations: BMI, body mass index; SBP, Systolic Blood Pressure; DBP, Diastolic Blood Pressure; LVEF, left ventricular ejection fraction; LVDD, left ventricular end diastolic diameter; E/e, the ratio of early mitral inflow velocity to early diastolic mitral annular velocity; NLR, neutrophil to Lymphocyte ratio; UA, uric acid; IVS, interventricular septum; LVPW, left ventricular posterior wall thickness. TC, total cholesterol; LDL-C, low density lipoprotein cholesterol; BNP, B-type natriuretic peptide; sST2, soluble suppression of tumorigenicity 2; eGFR, estimated glomerular filtration rate; CS, Cardiac bridging integrator 1 score; HFpEF, Heart Failure with Preserved Ejection Fraction.

Table S3. Sensitivity Analysis: Multivariate Logistic Regression of Independent Predictors for HFpEF After Excluding Patients with Renal Impairment

| **Variables** | **B** | | **SE** | | **Wald** | | ***P*** | | **OR** | | **95% CI**  **Lower Upper** | | |
| --- | --- | --- | --- | --- | --- | --- | --- | --- | --- | --- | --- | --- | --- |
| LVEF (%) | | -0.556 | | 0.153 | | 13.296 | | <0.001 | | 0.573 | | 0.425 | 0.773 |
| E/e' | | 3.210 | | 0.967 | | 11.024 | | 0.001 | | 24.785 | | 3.725 | 164.889 |
| NLR | | 0.398 | | 0.181 | | 4.839 | | 0.028 | | 1.489 | | 1.044 | 2.124 |
| BNP (pg/ml) | | 0.048 | | 0.015 | | 10.392 | | <0.001 | | 1.049 | | 1.019 | 1.080 |
| CS | | 2.517 | | 0.743 | | 11.474 | | 0.001 | | 12.388 | | 2.888 | 53.136 |

Abbreviations: LVEF, left ventricular ejection fraction; LVDD, left ventricular end diastolic diameter; E/e, the ratio of early mitral inflow velocity to early diastolic mitral annular velocity; NLR, neutrophil to Lymphocyte ratio; BNP, B-type natriuretic peptide; CS, cardiac bridging integrator 1 score.

Table S4. Sensitivity Analysis: Diagnostic Value of CS, BNP, Their Combinations, sST2, and CS Combined with sST2 for Cardiovascular Events in HFpEF Patients After Excluding Those with Renal Impairment

| **Variables** | **AUC** | ***P-*value** | **Sensitivity** | **Specificity** | **Cutoff value** | **Youden’s index** | **95% CI**  **Lower** | **95% CI**  **Upper** |
| --- | --- | --- | --- | --- | --- | --- | --- | --- |
| CS | 0.825 | <0.001 | 69.7% | 75.0% | 3.938 | 0.447 | 0.765 | 0.886 |
| BNP (pg/ml) | 0.913 | <0.001 | 82.0% | 94.8% | 103.5 | 0.768 | 0.870 | 0.957 |
| CS combined with BNP | 0.955 | <0.001 | 87.60% | 95.8% | 0.532 | 0.834 | 0.925 | 0.986 |
| sST2(ng/ml) | 0.784 | <0.001 | 69.7% | 80.2% | 11.300 | 0.499 | 0.715 | 0.852 |
| CS combined with sST2 | 0.825 | <0.001 | 60.7% | 93.7% | 0.607 | 0.544 | 0.765 | 0.886 |

Abbreviations: BNP, B-type natriuretic peptide; sST2, soluble suppression of tumorigenicity 2; CS, cardiac bridging integrator 1 score; HFpEF, Heart Failure with Preserved Ejection Fraction.

Table S5. Sensitivity Analysis: Predictive Performance of CS for MACE Occurrence After Excluding Patients with Renal Impairment

| **Variables** | **B** | **SE** | **Wald** | ***P*-value** | **OR** | **95% CI**  **Lower Upper** | |
| --- | --- | --- | --- | --- | --- | --- | --- |
| Stratification by CS | 1.389 | 0.527 | 6.952 | 0.008 | 4.011 | 1.428 | 11.261 |
| LVEF (%) | -0.114 | 0.050 | 5.231 | 0.022 | 0.892 | 0.809 | 0.984 |
| BNP (pg/ml) | 0.009 | 0.002 | 17.300 | <0.001 | 1.009 | 1.005 | 1.013 |

Abbreviations: LVEF, left ventricular ejection fraction; BNP, B-type natriuretic peptide;
